# A Case of Pancreatic Cancer in the Setting of Autoimmune Pancreatitis with Nondiagnostic Serum Markers

**DOI:** 10.1155/2013/809023

**Published:** 2013-05-28

**Authors:** Manju D. Chandrasegaram, Su C. Chiam, Nam Q. Nguyen, Andrew Ruszkiewicz, Adrian Chung, Eu L. Neo, John W. Chen, Christopher S. Worthley, Mark E. Brooke-Smith

**Affiliations:** ^1^Hepatobiliary Unit, Royal Adelaide Hospital, North Terrace, Adelaide, SA 5000, Australia; ^2^Department of Gastroenterology, Royal Adelaide Hospital, Adelaide, SA 5000, Australia; ^3^Department of Surgical Pathology, SA Pathology, Adelaide, Australia; ^4^Department of Gastroenterology, Flinders Medical Centre, Bedford Park, SA 5042, Australia

## Abstract

*Background*. Autoimmune pancreatitis (AIP) often mimics pancreatic cancer. The diagnosis of both conditions is difficult preoperatively let alone when they coexist. Several reports have been published describing pancreatic cancer in the setting of AIP. *Case Report*. The case of a 53-year-old man who presented with abdominal pain, jaundice, and radiological features of autoimmune pancreatitis, with a “sausage-shaped” pancreas and bulky pancreatic head with portal vein impingement, is presented. He had a normal serum IgG4 and only mildly elevated Ca-19.9. Initial endoscopic ultrasound-(EUS-) guided fine-needle aspiration (FNA) of the pancreas revealed an inflammatory sclerosing process only. A repeat EUS guided biopsy following biliary decompression demonstrated both malignancy and features of autoimmune pancreatitis. At laparotomy, a uniformly hard, bulky pancreas was found with no sonographically definable mass. A total pancreatectomy with portal vein resection and reconstruction was performed. Histology revealed adenosquamous carcinoma of the pancreatic head and autoimmune pancreatitis and squamous metaplasia in the remaining pancreas. *Conclusion*. This case highlights the diagnostic and management difficulties in a patient with pancreatic cancer in the setting of serum IgG4-negative, Type 2 AIP.

## 1. Introduction

Sarles et al. described the first reported case of idiopathic chronic pancreatitis with hypergammaglobulinemia secondary to an autoimmune process in 1961 [1]. It was, however, not until 1995 when Yoshida et al. proposed autoimmune pancreatitis (AIP) as a diagnostic entity [2]. AIP is characterised by unique clinical, radiological, and histological features. AIP commonly presents with obstructive jaundice and enlargement of the pancreas and mimics pancreatic cancer [3]. Several diagnostic criteria for AIP have been proposed. There are very few cases reported in the literature of both pancreatic cancer and autoimmune pancreatitis diagnosed together. Here, an unusual case of pancreatic cancer in the setting of autoimmune pancreatitis is reported, and the diagnostic and management dilemmas explored. 

## 2. Case Report

A 53-year-old man presented initially to his general practitioner with epigastric pain, jaundice, and significant weight loss over the preceding 3 months. He had no significant past medical or surgical history. He smoked 20 cigarettes a day and denied regular alcohol consumption. On examination, he was clinically jaundiced and had a soft abdomen with a nontender palpable gall bladder.

His initial blood tests demonstrated deranged liver function (Bili 160 umol/L, GGT 617 U/L, and ALP 649 U/L, ALT 342 U/L, AST 170 U/L), while his CA19-9 was only mildy raised at 81 kU/L (normal ref < 37). Serum IgG4 was not raised at 0.31 g/L (normal ref < 1.50). 

**Figure 1:**
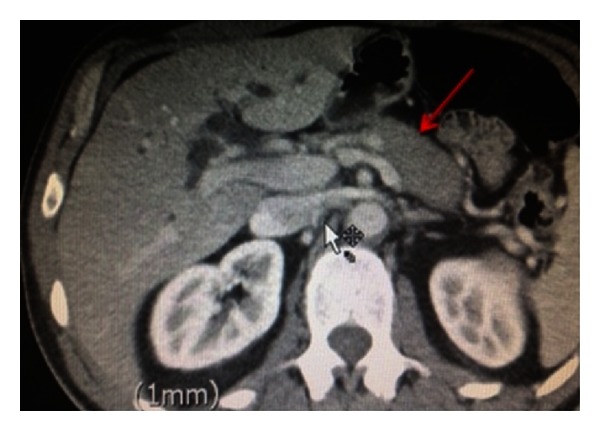
CT abdomen with “sausage-shaped” pancreas.

**Figure 2:**
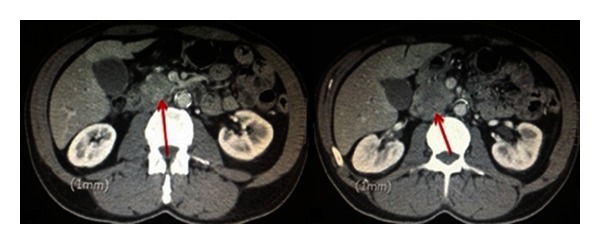
CT abdomen revealing a bulky pancreatic head.

**Figure 3:**
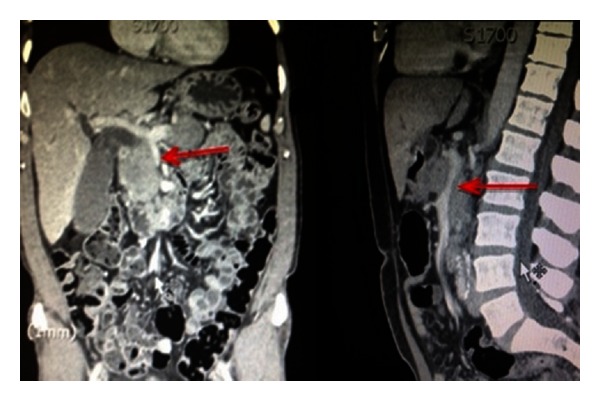
Portal vein narrowed with evidence of impingement on CT axial and sagittal images.

An ultrasound demonstrated biliary dilatation with no obvious mass. Subsequent computed tomography (CT) of his abdomen showed a bulky pancreatic head and “sausage-shaped” pancreas. His pancreatic duct was normal in calibre (Figures 1 and 2). The portal and superior mesenteric veins were significantly narrowed and impinged (Figure 3). The radiological features suggested autoimmune pancreatitis, though a malignant process could not be excluded.

**Figure 4:**
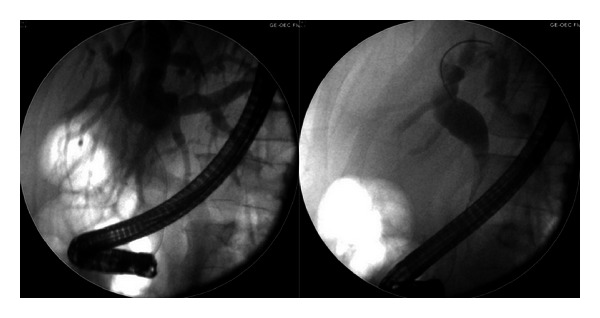
Endoscopic retrograde cholangiopancreatography (ERCP) images with dilated biliary tree down to a strictured distal bile duct.

He underwent endoscopic biliary decompression and placement of a biliary stent for his symptomatic jaundice. His intrahepatic and extrahepatic biliary tree was dilated to the lower third of the common bile duct (CBD), at which point the lower CBD appeared narrowed (Figure 4). 

**Figure 5:**
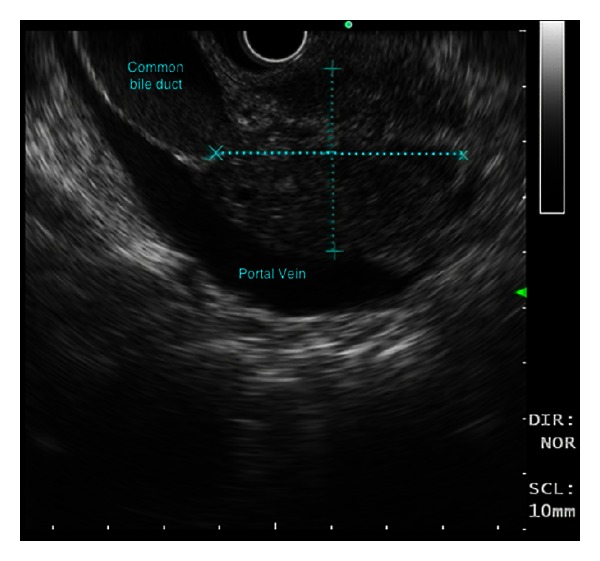
An irregular hypoechoic area within the head of the pancreas above, which the common bile duct tapered.

**Figure 6:**
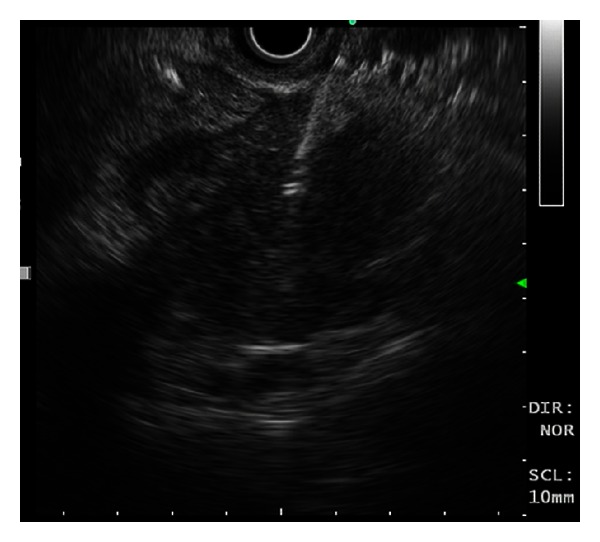
EUS-guided FNA of the pancreatic mass.

At time of ERCP, an endoscopic ultrasound (EUS) was performed. There was a 33 × 45 mm irregular hypoechoic area seen within the head of the pancreas, above which the CBD tapered (Figure 5). The pancreatic duct had a normal calibre in its course. There was also calibre change of the portal vein and superior mesenteric vein in the region of the pancreatic head without clear evidence of invasion. Hypoechoic nodes measuring 10 mm and 8 mm were seen at the peripancreatic head and celiac axis, respectively. EU-guided fine-needle aspiration (FNA) biopsy of the head of the pancreas performed revealed loosely fibrous stromal tissue with mildly distorted, reactive ductal epithelial element, and lymphocytic infiltration (Figure 6). Further FNA of the celiac and peripancreatic nodes showed reactive lymphoid cells. No malignant cells were identified in the biliary duct brushings nor in the FNA sample.

Whilst waiting for his jaundice to settle prior to planned resection given the clinical suspicion of a malignant process, a repeat EUS and FNA of the pancreatic head was performed. Similar appearances to the initial EUS were seen. Biopsies revealed features of autoimmune pancreatitis, and adenocarcinoma was revealed from one biopsy specimen.

At planned laporatomy, he had, an indurated, hard and diffusely enlarged pancreas without clear demarcation between the malignant tumour and autoimmune pancreatitis. There was no sonographically definable mass, raising concern that a pancreaticoduodenectomy may carry a risk of leaving tumour behind. Also, given the markedly enlarged gland, it would have been difficult to safely perform a pancreaticojejunostomy. With the positive preoperative pathology findings to guide, a total pancreatectomy with portal vein resection and partial superior mesenteric vein resection and reconstruction with an interposition graft of splenic vein was performed. 

**Figure 7:**
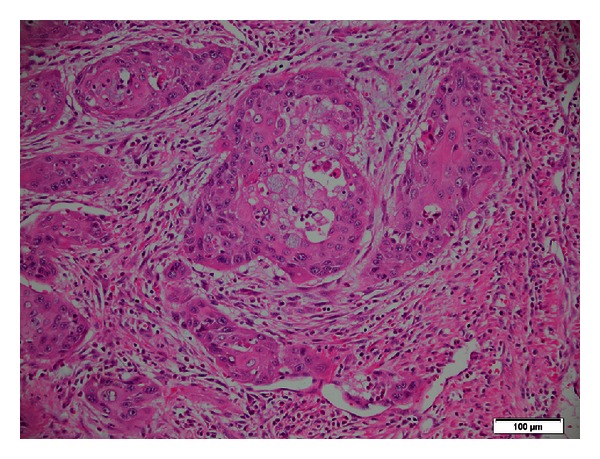
Histopathology revealed carcinoma with squamous differentiation and some admixed tumour cells containing intracytoplasmic mucin.

**Figure 8:**
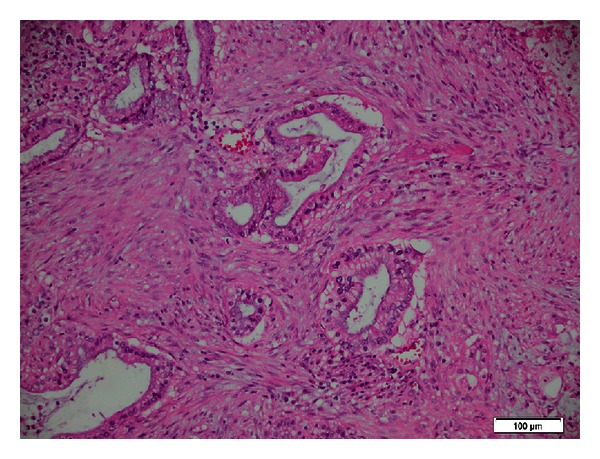
Histopathology of areas with gland formation (adenocarcinoma).

**Figure 9:**
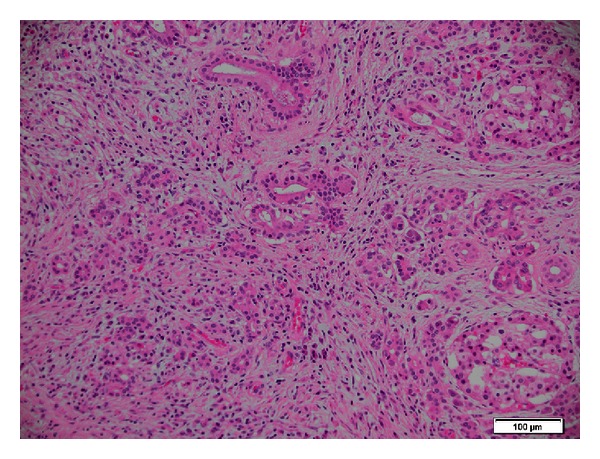
Histopathology showed inflammatory, sclerosing process involving benign region of pancreatic tissue. There was fibrosis atrophy of exocrine elements and lymphoplasmacytic inflammation. Residual pancreatic islets are also present.

Histology revealed a moderately differentiated 30 mm adenosquamous carcinoma of the head of the pancreas with invasion of the duodenal wall and metastases into 4 of 40 lymph nodes; margins were clear, and his portal vein was clear with extensive changes of autoimmune pancreatitis (Figures 7 and 8). The nonneoplastic portion of the pancreas showed extensive chronic pancreatitis with features consistent with autoimmune pancreatitis and squamous metaplasia (Figure 9).

The patient was heparinised postoperatively, and a Doppler ultrasound of his portal vein on the first postoperative day revealed good flow through the graft. He recovered well postoperatively with close management of blood sugar levels. He was discharged home 2 weeks postsurgery and is currently undergoing adjuvant chemotherapy. 

## 3. Discussion

Autoimmune pancreatitis (AIP) is a subset of chronic pancreatitis, which has been extensively reported from Japan, Europe, and USA. Diagnostic criteria for AIP include the Japan Pancreas Society Criteria [4], the Mayo Clinic's HISORt Criteria [5], the Korean criteria [6], and the Asian consensus criteria [7]. Descriptions from Japan have been predominantly based on the presence of distinct clinical phenotypes:

1. radiological imaging showing diffuse enlargement of the pancreas with diffuse irregular narrowing of the pancreatic duct,
2. elevated levels of serum gamma globulin or the presence of autoantibodies,
3. histopathological examination of pancreas showing periductal lymphoplasmacytic infiltration and fibrosis [4].

For a diagnosis of AIP, criteria 1 must be present, together with criterion 2 and/or 3.

Subsequent studies from Europe and USA have described 2 distinct histopathologic patterns of AIP. Type 1 AIP is lymphoplasmacytic sclerosing pancreatitis (LPSP) and has also been called IgG4-related pancreatitis due to the multiple extrapancreatic organ involvement of this disease [8]. Type 2 AIP is idiopathic duct-centric pancreatitis (IDCP) or granulocyteepithelial lesion-(GEL-) positive pancreatitis, which is rare in Japan and more prevalent in Europe and the USA. Clinically, the 2 variants present similarly and exhibit a dramatic response to steroids. Patients with Type 1 AIP tend to be older, with other organ involvement in 60% of patients [9]. Both types have distinct clinical profiles [10]. While Type 1 is associated with a raised IgG4 level, Type 2 is not [11]. However, 20% of patients with Type 1 AIP are seronegative [9].

The diagnosis of autoimmune pancreatitis in the early stages was guided by fulfilling the Japanese Pancreas Society's minimal consensus criteria. The Mayo Clinic in seeking to expand the diagnostic criteria proposed also looking for other organ involvement and response to steroid therapy in addition to the Japanese criteria—this is known as the HISORt criteria [5].

Clinically, AIP usually presents with upper abdominal pain, jaundice, and weight loss. Up to 50% of AIP presents with glucose intolerance [12]. Global pancreatic enlargement has been regarded as a main feature and has been described in the literature as “sausage-shaped” enlargement [13]. Our patient presented with upper abdominal pain, jaundice, and weight loss, similar presenting features of pancreatic cancer. His CT abdomen described a “sausage-shaped” enlargement of the pancreas. He also had a normal calibre pancreatic duct on ERCP. The Japanese diagnostic criterion for AIP requires demonstration of irregular narrowing of the main pancreatic duct on ERCP which he did not have [14]. 

EUS-guided FNA is a useful diagnostic tool to aid the diagnosis of pancreatic cancer. However, because of the small sample size obtained by this method, definitive diagnosis is sometimes difficult [14]. For this reason, EUS-guided core biopsy is recommended [15]. In this case, histology from the first EUS-guided FNA of the head of the pancreas showed loose fibrous stromal tissue with lymphocytic inflammation which was supportive of an inflammatory sclerosing process. The clinical suspicion of a neoplastic process led to a repeat EUS-guided FNA. This repeat FNA of the head of the pancreas, using a cellblock preparation technique, confirmed features of adenocarcinoma along with benign pancreatic tissue demonstrating a sclerosing pancreatitis pattern with many IgG4-positive cells. 

Differentiating pancreatic cancer from autoimmune pancreatitis can be difficult and requires a combination of clinical, serological, morphological, and histological features. Fluctuating obstructive jaundice, raised IgG4 levels, delayed enhancement of the pancreas, and capsule-like rim enhancement of the enlarged pancreas suggest AIP rather than pancreatic cancer. Presence of other organ involvement such as bilateral salivary gland swelling, retroperitoneal fibrosis, and hilar or intrahepatic sclerosing cholangitis also favours AIP. Pancreatic duct features of AIP on ERCP are >3 cm narrowing of main pancreatic duct, skip lesions of the main pancreatic duct, and <5 mm upstream dilatation of the main pancreatic duct [3, 16, 17]. Despite numerous features, in 30% of patients with AIP, the diagnosis cannot be confirmed without a steroid trial, pancreatic core biopsy, or surgical resection [15].

The patient presented represents an even more difficult group of patients who may have cancer in the setting of autoimmune pancreatitis. Several reports have been published describing pancreatic cancer in the setting of AIP [18–21]. Amongst the reports were a patient who developed metastatic carcinoma following a recent diagnosis of autoimmune pancreatitis and another elderly gentleman who developed pancreatic cancer 3 years following a diagnosis of AIP, for which steroid therapy induced a remission. A recent review assessing the risk of pancreatic cancer in the setting of autoimmune pancreatitis in 28 patients found that 82% of patients with autoimmune pancreatitis had pancreatic intraepithelial neoplasia (PanIN). Of 84 patients with AIP at their institution, there were 2 cases of pancreatic carcinoma at 6 and 10 years after diagnosis [22]. They concluded that AIP is associated with an increased risk of malignancy, and further studies are needed. This case along with other similar cases stresses the need for vigilance and close followup of patients in the setting of AIP.

## 4. Conclusion 

Autoimmune pancreatitis mimics pancreatic carcinoma clinically and radiologically. Though efforts have been made to develop strategies to distinguish between AIP and pancreatic cancer, cases like these represent a complex diagnostic dilemma. 

Serum markers of AIP and pancreatic cancer are not always helpful in aiding the diagnosis of both conditions, and EUS can be associated with sampling errors but offers potential for diagnostic information that greatly assists in operative and nonoperative decision making.
